# Prevalence and characteristics of sessile serrated lesions with dysplasia in Dutch fecal immunochemical test-positive screenees

**DOI:** 10.1055/a-2847-3376

**Published:** 2026-04-29

**Authors:** Nanette S. van Roermund, Valentina Angerilli, Iris D. Nagtegaal, Manon C.W. Spaander, Monique E van Leerdam, Joep E.G. IJspeert, Evelien Dekker

**Affiliations:** 1 Department of Gastroenterology and HepatologyAmsterdam University Medical Center, University of AmsterdamAmsterdamNetherlands; 2 571165 Amsterdam Gastroenterology Endocrinology MetabolismAmsterdamNetherlands; 3 Cancer Center AmsterdamAmsterdam University Medical CenterAmsterdamNetherlands; 4 Department of PathologyRadboud University Medical Centre, Radboud Institute for Molecular Life SciencesNijmegenNetherlands; 5 Department of Gastroenterology and Hepatology 6993 Erasmus MC University Medical Center RotterdamRotterdamNetherlands; 6 Department of Gastrointestinal OncologyNetherlands Cancer Institute – Antoni van Leeuwenhoek HospitalAmsterdamNetherlands; 7 Department of Gastroenterology and HepatologyLeiden University Medical CenterLeidenNetherlands; 8 Amsterdam Gastroenterology, Endocrinology and MetabolismAmsterdamNetherlands

## Abstract

**Background:**

Sessile serrated lesions (SSLs) with dysplasia (SSLDs) are considered high-risk polyps. We aimed to provide robust epidemiologic data on SSLDs over time in screening individuals with positive fecal immunochemical tests (FITs).

**Methods:**

All colonoscopy and pathology data of FIT-positive screenees in the Dutch colorectal cancer screening program (2014–2023) were analyzed. Prevalence of SSLs and SSLDs was calculated, along with number needed to scope (NNS). Dysplasia rate within SSLs was calculated over time. Multivariable logistic regression evaluated the influence of age and sex on SSLD prevalence, and assessed patient and polyp characteristics associated with dysplasia at polyp level.

**Results:**

Among 516 193 FIT-positive screenees, 1 033 298 polyps were removed, including 76 771 SSLs, of which 7010 (9.1%) exhibited dysplasia. Overall prevalence was 9.7% (95%CI 9.6–9.8) for SSLs and 1.1% (95%CI 1.1–1.1) for SSLDs. NNS to detect one SSL decreased over time, from 13 (2014–2018) to 9 (2019–2023), while NNS for SSLD remained stable at 90. The proportion of SSLDs declined from 17.5% in 2014 to 6.6% in 2023. Increasing age was associated with a higher SSLD prevalence (odds ratio per year 1.03; 95%CI 1.03–1.04) and dysplasia was more frequent in larger polyps (P < 0.001); however, 64.4% of SSLDs were <10 mm in size.

**Conclusions:**

This study showed a 1.1% prevalence of SSLD in FIT-positive screenees, offering valuable reference data. The finding that most SSLDs (64%) were small underscores the need to carefully assess small serrated polyps for dysplasia to ensure appropriate resection.

## Abstract (de)

**Graphical Abstract:**

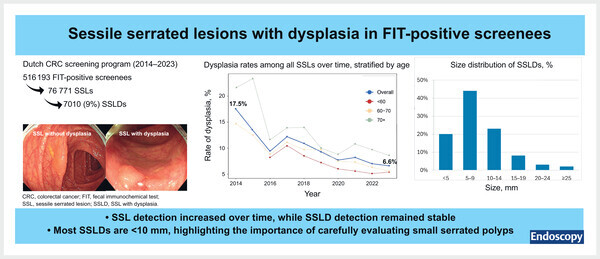

## Introduction

**Fig. 1:**
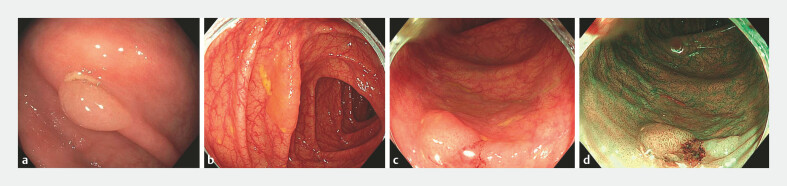
Endoscopic images. **a** Hyperplastic polyp. **b** Sessile serrated lesion without dysplasia. **c,d** Sessile serrated lesion with dysplasia under white light ( **c** ) and narrow-band imaging ( **d** ).

Colorectal cancer (CRC) was long believed to develop solely from adenomas via the adenoma–carcinoma pathway. In recent decades serrated polyps have also been recognized as precursors of CRC through the serrated neoplasia pathway 1 . Although our understanding of serrated polyps has grown as a result of substantial research in recent years, it remains less comprehensive than that of adenomas. Currently, serrated polyps are classified into four distinct subtypes according to the 2019 World Health Organization (WHO): 1) hyperplastic polyps, 2) sessile serrated lesions (SSLs), which can be subcategorized into SSLs without dysplasia and SSLs with dysplasia (SSLDs) ( Fig. 1 ), 3) traditional serrated adenomas, and 4) serrated adenomas unclassified 2 .

SSLs are considered the main precursor lesions of CRC, progressing through an intermediate stage characterized by a dysplastic focus 1 . Research suggests that SSLs have a long dwell time, approximately 20 years, before progressing to SSLDs 3 . However, once dysplasia develops within SSLs, these lesions can progress rapidly into CRC, in some cases in less than 1 year according to estimates 3 4 . Together, these observations emphasize the importance of distinguishing between SSLs with and without dysplasia for risk stratification, surveillance planning, and the prevention of CRC.

The relatively recent recognition of SSLs and SSLDs has led to substantial interobserver variability among endoscopists and pathologists 5 6 . This, combined with the rarity of SSLDs and the small sample sizes in existing studies evaluating risk factors, has resulted in limited and inconsistent prevalence estimates 7 8 9 . As a result, reliable data on the prevalence and risk profile of SSLDs in screening populations are still lacking. Robust evidence is urgently needed to support endoscopists in detection and management, to help policymakers and screening programs to refine guidelines and quality assurance programs, and to provide researchers with reliable reference data for future studies. To fill this important knowledge gap, we conducted a large epidemiologic study aiming to provide accurate prevalence estimates and identify factors positively associated with SSLDs in screenees with a positive fecal immunochemical test (FIT).

## Methods

### Study design and population

This population-based study utilized prospectively collected colonoscopy data from FIT-positive screenees in the Dutch national CRC screening program, covering a 10-year period from January 2014 to December 2023. In the Netherlands, all inhabitants aged 55–75 years are biennially invited to undergo an FIT. Those with a positive FIT result are subsequently referred for colonoscopy. The FIT cutoff for positivity was initially set at 15 µg hemoglobin (Hb) per gram feces when the screening program was implemented in 2014, but after 6 months the cutoff was increased to 47 µg Hb/g feces 10 . The screening program in the Netherlands was implemented in a phased manner, with successive age cohorts invited each year until, by 2019, all individuals aged 55–75 years were invited. Consequently, the first few years of the program included participants with a higher median age.

Endoscopists performing colonoscopies for FIT-positive screenees in the Dutch CRC screening program are required to meet specific quality standards. Accreditation standards include performing at least 200 colonoscopies per year, conducting at least 50 polypectomies per year, achieving a cecal intubation rate of over 95%, maintaining an adenoma detection rate of at least 40%, and removing at least 90% of detected polyps 11 . The quality indicators for both the endoscopist and the colonoscopy procedure are continuously monitored and are audited annually.

To maintain the quality of pathology assessments, pathologists involved in the screening program are regularly audited at the case level, as well as having to take part in national slide seminars and being required to follow specific educational sessions. Since 2014, they have also been required to complete a validated e-learning course on the histopathologic diagnosis of serrated polyps before being allowed to participate in pathology reporting for the screening program 12 .

### Ethical approval

Given the retrospective design of this study and the fact that individuals only received the standard of care, the Dutch Act on Medical Research Involving Human Subjects did not apply. Participant privacy was ensured by using pseudonyms before data transfer, in accordance with the General Data Protection Regulation Act. Returning the FIT was considered as consent for using pseudonymized data from all screening colonoscopies and pathology reports. All individuals retained the right to object to the use of their data.

### Inclusion and exclusion criteria

To determine the prevalence of SSLs and SSLDs in FIT-positive screenees and to assess SSLD characteristics, we evaluated the colonoscopy result for each FIT-positive screenee in conjunction with the corresponding pathology reports. If multiple colonoscopies were performed within the setting of the FIT screening program, only the first complete baseline colonoscopy with adequate bowel preparation (e.g. Boston Bowel Preparation Scale score of 2 in every segment) was included.

### Data collection

Data from all colonoscopies were obtained from the Dutch CRC screening organization (BVO NL), which collects information on colonoscopies performed in FIT-positive screenees within the Dutch CRC screening program. Data on all histopathologic diagnoses of polyps were retrieved (20 230 102) from the Dutch national pathology databank, known as Palga. Linkage between data from BVO and Palga was achieved using citizen service numbers. After pseudonymization of all combined data, we obtained a comprehensive dataset containing both colonoscopy and corresponding pathology data.

### Outcome definitions

The prevalence of SSLs and SSLDs within FIT-positive screenees was defined as the proportion of FIT-positive screenees in which at least one SSL or SSLD was detected, divided by the total number of screenees included. Prevalence estimates were reported as percentages with 95%CIs. Number needed to scope (NNS) was defined as the inverse of SSL or SSLD prevalence, representing the number of FIT-positive screenees who must undergo colonoscopy to detect at least one SSL or SSLD.

**Fig. 2:**
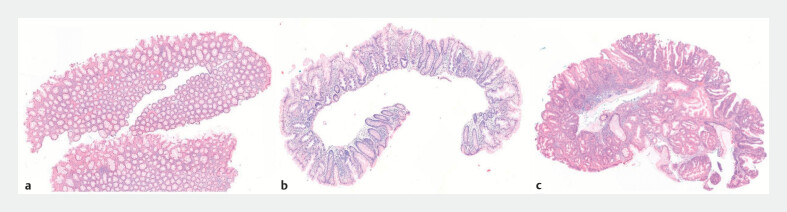
Histologic analysis. **a** Serration confined to the upper half of the crypts, which is characteristic of a hyperplastic polyp. **b** Serration extending into the lower third of the crypts, with inverted L- or T-shaped crypts above the muscularis mucosae and dilation of the crypt base, features characteristic of a sessile serrated lesion (SSL). **c** Typical SSL features, including dilated, boot-shaped crypts, as well as a transition to an area with cytologically dysplastic cells, which is characteristic of an SSL with dysplasia.

Pathologic diagnoses followed WHO recommendations ( Fig. 2 ) 2 . Hyperplastic polyps are characterized by elongated, straight crypts with serration limited to the upper half of the crypts. Before 2019, SSLs were distinguished from hyperplastic polyps by the presence of at least two of the following features in two noncontiguous crypts: 1) serration in the lower third, 2) L- or T-shaped crypts above the muscularis mucosae, 3) inverted crypts above the muscularis mucosae, and 4) columnar dilation in the lower third of the crypts. In 2019, the criteria were updated such that a single unequivocal SSL crypt was sufficient for diagnosis. SSLDs exhibit unequivocal cytologic and/or architectural features of dysplasia in an SSL. Per WHO recommendations, dysplasia in SSLs is not graded as low or high grade; only the term “SSLD” is used without further classification of dysplastic patterns.

Lesion size (measured in mm) was determined based on the histopathologic specimen and categorized into six categories (<5, 5–9, 10–14, 15–19, 20–24, ≥25 mm). The proximal colon was defined as the region proximal to the descending colon, including the splenic flexure. Dysplasia rate of SSLs was defined as the proportion of SSLDs among all SSLs.

### Statistical analysis

Prevalence of SSLs and SSLDs within FIT-positive screenees was calculated for the entire study period and stratified by age categories (<60, 60–70, >70) and sex. Prevalence was calculated as proportion with 95%CIs based on the Wilson score method for binominal data. To assess trends over time, prevalence within FIT-positive screenees was calculated separately for two study periods: 2014–2018 and 2019–2023. Additionally, to illustrate changes in SSL and SSLD prevalence over time, the NNS was calculated for each time period and presented graphically.

Baseline characteristics were analyzed at both FIT-positive screenee level (age, sex), and SSL level (location, age category, and size category). Descriptive statistics were used to summarize categorical variables as counts with proportions and continuous variables as median with interquartile range (IQR). Categorical variables were compared using the chi-squared test. Continuous variables were assessed for normality and compared using the Mann–Whitney *U* test when non-normally distributed. Dysplasia rates of SSLs were calculated for each polyp characteristic and for each year of colonoscopy to assess temporal trends.

At the FIT-positive screenee level, univariable and multivariable logistic regression analyses were performed to assess characteristics associated with presence of an SSL or SSLD. The multivariable model was adjusted for age, sex, and year of colonoscopy, to account for potential time trends. Results of the logistic regression models were reported as odds ratios (OR) with 95%CI.

To adjust for age at the SSL level, patient age from the FIT-positive screenee dataset was linked to the polyp dataset using patient pseudo ID, and each SSL was then assigned to an age category. Because multiple polyps could be observed within the same patient, univariable and multivariable logistic regression with generalized estimating equations was used, specifying patient pseudo ID as the clustering variable, with dysplasia (yes/no) as the dependent variable to assess characteristics (location, polyp size, age category) associated with dysplasia in SSLs. The multivariable model was adjusted for significant subgroups identified in univariable analysis, with results reported as OR with 95%CI. All statistical tests were two-sided, and a *P* value <0.05 was considered statistically significant. Analyses were performed using R-studio (R Foundation for Statistical Computing, Vienna, Austria).

## Results

### Baseline characteristics

Between January 2014 and December 2023, 516 193 screenees had positive FIT results. The median age was 65 (IQR 61–71) years and 56.6% were male. Among these individuals, 1 033 298 polyps were removed, 229 165 (22.2%) of which were serrated and 804 133 (77.8%) were adenomas. Among the removed serrated polyps, 76 771 (33.5%) were SSLs, of which 7010 (9.1%) exhibited dysplasia, 146 943 (64.1%) were hyperplastic polyps, and 5451 (2.4%) were traditional serrated adenomas. Of the 7087 serrated polyps with dysplasia, for which size was available, 1013 (14.3%) measured <5 mm in size and 3521 (49.7%) were <10 mm. Among the removed adenomas, 680 417 (84.6%) were tubular adenomas, 117 649 (14.6%) were tubulovillous adenomas, 6067 (0.8%) were villous adenomas, and 17 482 (2.2%) exhibited high grade dysplasia (HGD). Of the 11 975 adenomas with HGD, for which polyp size were available, 175 (1.5%) measured <5 mm in size and 1896 (15.8%) were <10 mm. The co-occurrence of polyps in individuals with SSLs versus SSLDs is presented in **Table 1s** in the online-only Supplementary Material.

### Prevalence and NNS of SSLs and SSLDs

In total, 49 953 individuals had at least one SSL, of whom 5737 had at least one SSLD. During the total study period of 10 years, the prevalence of SSLs in FIT-positive screenees was 9.7% (95%CI 9.6–9.8), while the prevalence of SSLDs in FIT-positive screenees was 1.1% (95%CI 1.1–1.1).

Within the entire cohort, SSL prevalence in FIT-positive screenees was higher in females than in males (male as reference OR 1.07, 95%CI 1.05–1.09), while sex was not associated with SSLD prevalence (male as reference OR 1.05, 95%CI 0.99–1.11). The median age of individuals with SSLs was 65 years (IQR 61–71), while the median age of individuals with SSLDs was 67 years (IQR 63–72) ( *P* < 0.001). No association was observed between age and SSL prevalence (OR 0.99 per year increase, 95%CI 0.99–1.00), whereas increasing age was clearly associated with SSLD prevalence in FIT-positive screenees (OR 1.03 per year increase, 95%CI 1.03–1.04).

During the 10-year study period, SSL prevalence steadily increased (OR per increasing colonoscopy year 1.09, 95%CI 1.08–1.09), while SSLD prevalence in FIT-positive screenees showed no significant change over time (OR per increasing colonoscopy year 0.99, 95%CI 0.98–1.01).

**Fig. 3:**
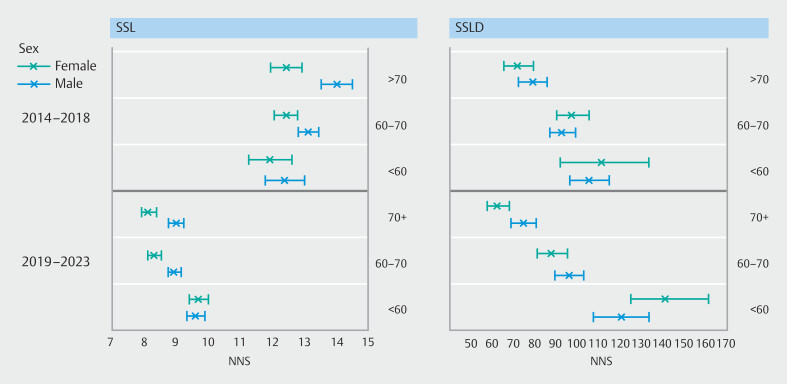
Number needed to scope (with 95%CI) for sessile serrated lesions and sessile serrated lesions with dysplasia over time, stratified by age and sex. NNS, number needed to scope; SSL, sessile serrated lesion; SSLD, sessile serrated lesions with dysplasia.

To illustrate these trends, Fig. 3 presents the age-stratified prevalence of SSLs and SSLDs in FIT-positive screenees across two time periods, expressed as NNS. The corresponding prevalences within FIT-positive screenees are presented in **Table 2s** and **Table 3s** . A clear temporal trend was observed for SSLs: the overall NNS decreased from 12.9 (95%CI 12.7–13.1) in 2014–2018 to 8.9 (95%CI 8.8–9.0) in 2019–2023. However, the overall NNS for SSLDs remained stable at 90. The lowest NNS for SSLDs was observed in females aged >70 years (63; 95%CI 58–68), while the highest was in females aged <60 years (141; 95%CI 125–161).

**Fig. 4:**
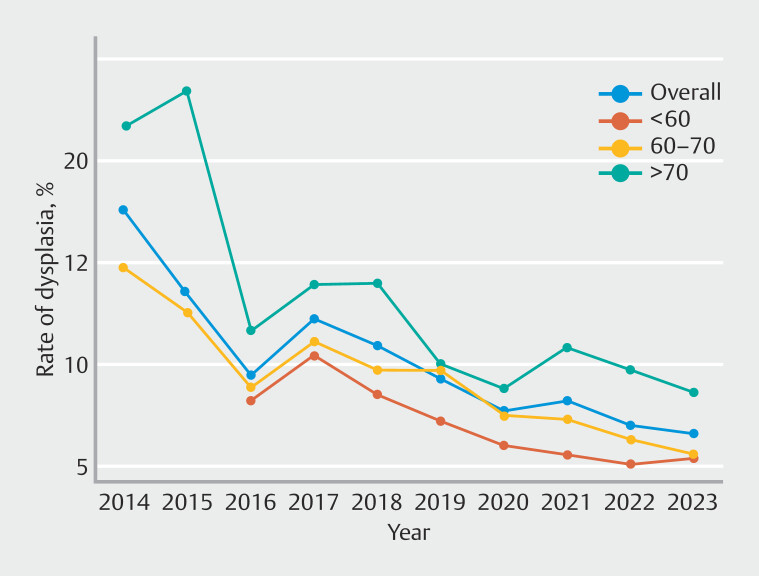
Rate of dysplasia of sessile serrated lesions over time stratified by age category.

Fig. 4 depicts the rate of dysplasia within SSLs over time, stratified by age category. The highest overall dysplasia rate was 17.5% in 2014. This rate declined to 9.4% in 2016 and since 2017, a steady decrease in the dysplasia rate was observed across all age categories, reaching the lowest overall dysplasia rate in 2023 (6.6%) ( **Table 4s** ).

### Characteristics of SSLs and SSLDs

Table 1 presents characteristics of SSLs and SSLDs on a per-polyp level. An increasing trend in rate of dysplasia was observed for advancing age ( *P* <0.001). SSLs in FIT-positive screenees aged >70 years demonstrated an OR of 1.79 (95%CI 1.64–1.95) for harboring dysplasia compared with SSLs in individuals aged <60 years.

**Table:** **Table 1** Polyp characteristics associated with dysplasia in sessile serrated lesions.

Per polyp	SSL N = 76 771, n (%)	SSLD N = 7010, n (%)	Dysplasia rate, % (n/N)	Univariable OR (95%CI)	*P* value	Multivariable OR (95%CI)	*P* value
Location, n (%)							
Cecum	10 978 (14.3)	841 (12.0)	7.7 (841/10 978)	0.82 (0.76–0.89)	<0.001	0.74 (0.68–0.80)	<0.001
Ascending colon	24 106 (31.4)	2264 (32.3)	9.4 (2264/24 106)	1.04 (0.99–1.10)	0.13	–	–
Hepatic flexure	845 (1.1)	63 (0.9)	7.5 (63/845)	0.81 (0.62–1.04)	0.11	–	–
Transverse colon	15 047 (19.6)	1410 (20.1)	9.4 (1410/15 047)	1.03 (0.97–1.09)	0.39	–	–
Splenic flexure	3070 (4.0)	315 (4.5)	10.3 (315/3070)	1.11 (0.99–1.25)	0.08	–	–
Descending colon	5067 (6.6)	484 (6.9)	9.6 (484/5067)	1.05 (0.95–1.16)	0.30	–	–
Sigmoid	11 362 (14.8)	1016 (14.5)	9.0 (1016/11 362)	0.97 (0.91–1.04)	0.42	–	–
Rectum	3992 (5.2)	365 (5.2)	9.1 (365/3992)	1.01 (0.90–1.12)	0.91	–	–
Rectosigmoid	1536 (2.0)	161 (2.3)	10.5 (161/1536)	1.15 (0.97–1.35)	0.10	–	–
Proximal	54 046 (70.4)	4893 (69.8)	9.0 (4893/54 046)	–	–	–	–
Distal	21 957 (28.6)	2026 (28.9)	9.2 (2026/21 957)	–	–	–	–
Undefined	768 (1.0)	91 (1.3)	11.8 (91/768)	–	–	–	–
Age in years, %							
<60	15 738 (20.5)	1009 (14.4)	6.4 (1009/15 738)	ref	–	ref	–
60–70	38 309 (49.9)	3358 (47.9)	8.8 (3358/38 309)	1.34 (1.24–1.46)	<0.001	1.28 (1.17–1.39)	<0.001
>70	22 724 (29.6)	2643 (37.7)	11.6 (2643/22 724)	1.82 (1.67–1.97)	<0.001	1.79 (1.64–1.95)	<0.001
Size in mm, % ^1^							
<5	13 290 (26.9)	700 (20.3)	5.3 (700/13 290)	ref	–	ref	-
5–9	24 247 (49.1)	1521 (44.1)	6.3 (1521/24 247)	1.20 (1.10–1.32)	<0.001	1.21 (1.11–1.33)	<0.001
10–14	8995 (18.2)	786 (22.8)	8.7 (786/8995)	1.72 (1.55–1.91)	<0.001	1.71 (1.54–1.90)	<0.001
15–19	2065 (4.2)	284 (8.2)	13.8 (284/2065)	2.87 (2.47–3.32)	<0.001	2.64 (2.27–3.08)	<0.001
20–24	523 (1.1)	94 (2.7)	18.0 (94/523)	3.94 (3.10–4.97)	<0.001	3.58 (2.78–4.61)	<0.001
≥25	247 (0.5)	60 (1.7)	24.3 (60/247)	5.77 (4.24–7.46)	<0.001	5.22 (3.74–7.29)	<0.001
OR, odds ratio; SSL, sessile serrated lesion; SSLD, sessile serrated lesion with dysplasia. ^1^ Size in mm was missing for 27 404 (35.7%) of SSLs and 3565 (50.9%) of SSLDs.							

In addition, an increase in size of the SSLs was associated with a higher rate of dysplasia ( *P* < 0.001). The dysplasia rate was 5.3% for SSLs <5 mm, increasing to 24.3% for SSLs ≥25 mm. Multivariable analysis showed that SSLs ≥25 mm had an OR of 5.22 (95%CI 3.74–7.29) for harboring dysplasia, compared with SSLs <5 mm. However, 64.4% of SSLDs had a size of <10 mm.

**Fig. 5:**
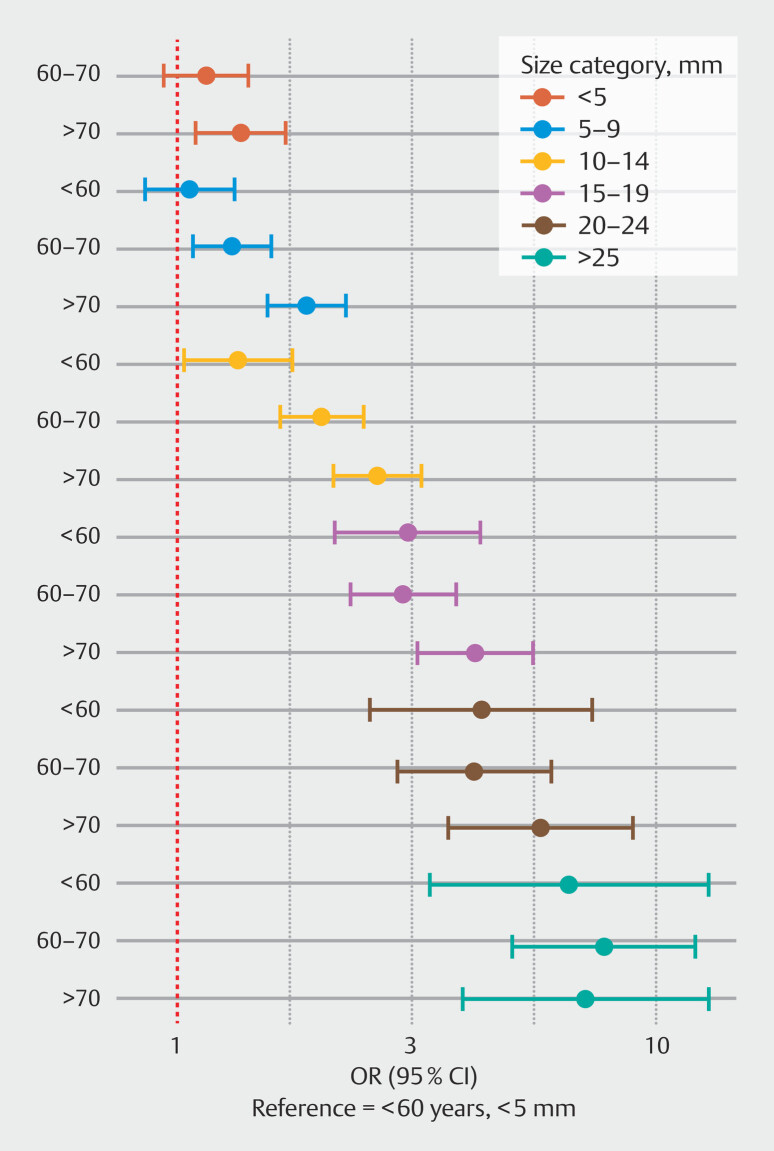
Odds ratio for dysplasia in sessile serrated lesions, stratified by age and size. OR, odds ratio.

ORs for rate of dysplasia within SSLs are presented in Fig. 5 , combining both age and size category. The odds of finding dysplasia in an SSL of at least 25 mm within individuals aged 60–70 years and >70 years were seven times higher (OR 7.73, 95%CI 4.29–11.9 for 60–70 years; OR 7.01, 95%CI 3.81–12.5 for >70 years) compared with the odds for individuals <60 years with an SSL <5 mm in size.

Both SSLs and SSLDs were predominantly located in the proximal colon (70%). A significant difference in colonic distribution was observed between SSLs and SSLDs ( *P* = 0.004). However, the absolute differences in percentages were minimal ( **Fig. 1s** ). Multivariable analysis demonstrated a slightly lower OR for SSLDs to be located in the cecum (OR 0.74, 95%CI 0.68–0.80).

## Discussion

In this large study, which comprised 10 years of colonoscopy data from over half a million Dutch FIT-positive screenees, we found an overall SSL prevalence of 9.7% (95%CI 9.6–9.8) and SSLD prevalence of 1.1% (95%CI 1.1–1.1). As the number of SSL diagnoses increased over time, while SSLD diagnoses remained stable, the rate of dysplasia in SSLs gradually declined from 17.5% in 2014 to 6.6% in 2023. Prevalence of SSLDs in FIT-positive screenees was associated with older age, but not with sex. The likelihood of dysplasia in SSLs increased with higher age and polyp size. SSLs of ≥25 mm detected in individuals >70 years of age had sevenfold higher odds of harboring dysplasia compared with SSLs <5 mm in individuals <60 years of age. However, a substantial 64% of SSLDs were <10 mm in size.

The findings of this study have several important clinical and public health implications. For endoscopists, the strong association between dysplasia, polyp size, and patient age provides a basis for risk stratification during colonoscopy and may guide decisions regarding resection techniques. For policymakers and quality assurance programs, these robust nationwide data provide a more accurate estimate of the true prevalence of SSLs and SSLDs in FIT-positive screenees. This information is vital for establishing reliable quality indicators related to serrated polyps, ultimately aiming to lower the incidence of post-colonoscopy CRC 13 and preserve the quality of CRC screening programs. Furthermore, these data may help refine guideline recommendations, particularly when recognizing the fact that a substantial proportion of SSLDs were <10 mm in size, underscoring that small SSLs can also carry clinical significance. Finally, for researchers, this study addresses a major evidence gap by presenting the largest dataset to date on SSLDs, offering a benchmark for future population-based studies and international comparisons. Together, these results highlight the importance of standardized definitions and diagnostic practices in reliably assessing the prevalence and biological relevance of SSLDs.

Reported SSL prevalence varies widely (0–16%) 14 due to evolving clinical and pathologic definitions 2 , advancements in endoscopic technology, and significant interoperator variability among both endoscopists and pathologists 5 6 . Since 2020, results from more standardized, population-based studies have emerged, reporting SSL prevalence between 4% and 6% 15 16 . Our observed SSL prevalence of 9.7% overall and 11.2% between 2019 and 2023 among FIT-positive screenees is notably higher. This elevated rate could, in part, be the result of a rigorous quality assurance program in the Netherlands, including strict endoscopist accreditation and standardized pathology training (e.g. SSL-focused e-learning modules 12 ). Alternatively, the FIT-based selection may also contribute to this higher yield compared with primary colonoscopy screening, as serrated polyps are frequently found alongside adenomas ( **Table 1s** ). However, as the literature shows no substantial difference in SSL detection rates between FIT-based and primary screening cohorts 16 17 , these comparisons remain valid. In contrast to SSL prevalence, the prevalence of SSLDs remains poorly defined within the literature. Only a few studies have reported the prevalence of SSLDs, with estimates ranging from 0.3% to 0.6% 18 19 . However, these estimates are based on small sample sizes, with the largest study including 553 patients with SSLDs. This stands in marked contrast to the current study, which comprised 5737 patients with SSLDs. In addition, larger studies often used broader definitions, such as “high-risk serrated polyps” or “clinically significant” serrated polyps 16 20 , making it difficult to estimate SSLD prevalence specifically. In our study, large-scale linkage of colonoscopy and pathology records, together with trained endoscopists and pathologists enabled a more precise estimate of SSLD prevalence of 1.1% among FIT-positive screenees. These findings may better reflect the true prevalence of SSLD.

Over the 10-year study period, dysplasia rates in SSLs steadily declined, from 17.5% in 2014 to 6.6% in 2023. Earlier studies reported widely varying dysplasia rates in SSLs, ranging from 0.9% to nearly 31% 7 18 21 , but these were predominantly older, single-center cohorts. The higher dysplasia rate observed during the early years of the program may be partly explained by the phased implementation of the Dutch screening program, in which a relatively large proportion of older individuals were invited first, before the program was fully rolled out to all individuals aged 55–75 years. However, dysplasia rates continued to decline even after full implementation of the screening program in 2019. Another possible explanation for the decreasing dysplasia rate is that nondysplastic SSLs may have been more frequently missed in the earlier years, whereas detection of SSLDs may already have been adequate throughout the entire study period. This would result in a relative decline in the proportion of dysplastic lesions over time. A potential reason for this earlier stabilization in SSLD detection is that SSLDs may be easier to recognize endoscopically, as dysplastic areas often display nodular or more pronounced vascular features 22 23 . The rise in SSL diagnoses since 2014 is likely driven by increased awareness and advances in endoscopic imaging. A third explanation may be that, in the 2019 WHO histopathologic criteria for diagnosing SSL, the presence of just one crypt with SSL features was sufficient for the diagnosis, whereas the earlier definition required at least two such crypts. This change has likely resulted in lesions that were previously classified as hyperplastic polyps being reclassified as SSLs 2 . Consistent with this, the proportion of hyperplastic polyps in the Dutch screening program decreased from 79% in 2014 to 54% in 2023. This marked decline in hyperplastic polyp diagnoses likely accounts for a substantial part of the concurrent increase in SSL diagnoses. The more recent stabilization in SSL diagnoses since 2021 ( **Table 4s** ) suggests that the dysplasia rate of 6.6% observed in 2023 more closely reflects the true dysplasia rate in SSLs. That said, some degree of underestimation of SSLD diagnoses cannot be ruled out. Misclassification as adenomas or nondysplastic SSLs may occur, and incomplete resection during endoscopy can further hinder accurate pathologic assessment 24 .

Earlier studies investigating the association between prevalence of SSLs or SSLDs and sex have shown conflicting results 25 26 . In our study, although SSLs were more prevalent in females, for SSLDs no such association was observed. This finding aligns with more recent studies that also reported no significant differences between sexes for SSLDs 18 27 . Regarding colonic location, both SSLs and SSLDs were predominantly located in the proximal colon (70%) 22 . This contrasts with several earlier studies, mainly from Asian populations, which reported that SSLDs were either evenly distributed throughout the colon or more frequently located distally, resembling the distribution pattern of adenomas 21 28 29 . These differences in localization may reflect ethnic or regional variation in SSL biology, but methodologic issues, such as limited sample size, could also play a role. Based on a Western cohort of over 7000 SSLDs, our study did not confirm these observations and instead demonstrated a predominantly proximal distribution for both SSLs and SSLDs.

In line with previous studies, this study confirms that increasing age is significantly associated with a higher risk of dysplasia in SSLs 22 26 . Individuals with SSLDs were on average 2 years older than those with nondysplastic SSLs (67 vs. 65 years). In 2017, Bettington et al. 3 reported a mean age of 75 years for individuals with SSLD, compared with 58 years for those with nondysplastic SSLs. Notably, the mean age at CRC diagnosis closely corresponded to that of the SSLD group. Based on these observations, they hypothesized a long dwell time of approximately 17 years for SSLs, followed by rapid malignant transformation once dysplasia develops. Our study did not support this hypothesis, as the age difference between individuals with SSLs and SSLDs in our cohort was less pronounced. This discrepancy between our findings and those of Bettington et al. may be attributed to the higher median age of the Dutch screening population. Nonetheless, Fig. 3 demonstrated a lower NNS for SSLD among older patients, while no such decrease was observed for SSLs, potentially reflecting the longstanding presence of SSLs prior to progression. In this cohort, information on the molecular origins of CRC was not available, therefore we could not assess the short dwell time from SSLD to cancer reported by Bettington et al. and other case reports 4 .

In adenomas, larger size is a well-established risk factor for HGD 30 . Similar associations have been identified for SSLs, with several studies reporting an increased likelihood of dysplasia in larger lesions 7 23 . Ohki et al. 7 and Murakami et al. 23 both demonstrated that the proportion of SSLDs rises with increasing size of the SSLs, a finding confirmed by our study. Interestingly, unlike adenomas, where small lesions rarely harbor HGD (15.8% of adenomas with HGD in this cohort were <10 mm in size), a substantial proportion of SSLDs appear to be small. Bettington et al. 3 reported that 54% of SSLDs were <10 mm, a finding later supported by Murakami et al. in 2024 23 , who found that 43% of SSLDs with HGD were <10 mm in size. Our study aligns with these observations, showing that 64% of SSLDs measured <10 mm. This highlights the relevance of recognizing dysplasia in both large SSLs and small SSLs, the latter being inherently more difficult to identify endoscopically. Several endoscopic features have been proposed as indicative of SSLD, including surface redness, a nodular appearance, mixed Kudo pit patterns, and JNET type 2 22 23 . Nonetheless, large-scale studies validating these features across a broad range of endoscopists are still lacking. Given that the clinical management of SSLDs differs from that of SSLs, most notably due to the need for an en bloc resection to mitigate the risk of post-colonoscopy CRC from a non-radically resected SSLD 31 , it would be valuable to develop standardized criteria for identifying SSLDs during endoscopy. This becomes even more important if future “resect-and-discard” strategies are implemented, as accurate recognition of small SSLDs would be critical in that context.

This study has several strengths. Most notably, the population-based design allowed for the inclusion of a large cohort of SSLDs by linking colonoscopy reports to pathology data. Given the rarity of SSLDs, assembling a sufficiently large sample is often challenging, limiting the generalizability of earlier findings. Additionally, the involvement of highly trained endoscopists and pathologists within the national screening program likely contributes to a more accurate estimation of the true prevalence of SSLs and SSLDs within FIT-positive screenees. However, this study also has several limitations. First, annotated endoscopic images and morphologic classifications (e.g. Paris classification) were not available in the dataset, preventing systematic evaluation of lesion-specific visual characteristics. Second, polyp size was recorded by the pathologists rather than endoscopists. For larger polyps resected piecemeal, however, it is common practice for pathologists to include the endoscopist’s size estimate, as provided in the request form, in their pathology reports. This explains why size data are available for these polyps. Third, although endoscopists and pathologists were subject to strict quality monitoring within the screening program, no centralized review of serrated polyps was performed. This may have introduced interobserver variability, potentially influencing the results.

Finally, although overall SSL prevalence appears comparable between primary screening and FIT-based settings, SSLD prevalence may differ. The vascular nature of dysplastic SSL components could lead to higher SSLD detection in FIT-based settings, potentially limiting the generalizability of our findings.

In conclusion, this cohort of individuals with SSLDs is the largest published to date and provides a realistic, population-based estimate of SSL (9.7%) and SSLD (1.1%) prevalence within FIT-positive screenees. The declining dysplasia rates in SSLs over time likely reflect the increasing recognition of SSLs by endoscopists and pathologists. The data from this study can offer clinicians, researchers, and policymakers a robust reference point for shaping future research priorities and screening policies. Given that 64% of SSLDs measured <10 mm, we emphasize the importance of endoscopists carefully evaluating small serrated polyps for potential dysplasia to ensure timely and appropriate treatment.

## Data transparency statement

Study data are available upon request to Professor Evelien Dekker.

## Supplementary Material

Supplementary Material Zusatzmaterial
