# Supplementary material for: Prevalence and characteristics of sessile serrated lesions with dysplasia in Dutch fecal immunochemical test-positive screenees
Source: Endoscopy. 2026 Apr 29;58(7):776–85. doi: 10.1055/a-2847-3376 (PMC13305575; doi:10.1055/a-2847-3376)

Supplementary material

Prevalence and characteristics of sessile serrated lesions with dysplasia in Dutch fecal immunochemical test-positive screenees

Nanette S. van Roermund, Valentina Angerilli, Iris D. Nagtegaal, Manon C.W. Spaander, Monique van Leerdam, Joep. E. G. Ijspeert, Evelien Dekker

Table 1s Co-occurring polyps in individuals with sessile serrated lesions versus sessile serrated lesions with dysplasia.

**Per individual**	**SSL N=49.953** Overall	**SSLD N=5.737** Overall	**SSL N=21.975** 2021-2023	**SSLD N=2.055** 2021-2023
**Co-occurring polyps, n** (%)				
≥1 adenoma	36.116 (72.2)	4.424 (77.1)	15.102 (68.7)	1.547 (75.3)
≥1 advanced adenoma*	16.697 (33.4)	2.214 (38.6)	5.539 (25.2)	622 (30.3)
≥1 high-risk adenoma*	15.807 (31.6)	2.075 (36.1)	5. 502 (25.0)	626 (30.5)
≥1 10mm adenoma	12.425 (24.9)	1.586 (27.6)	3.966 (18.0)	433 (21.1)
≥1 adenoma with HGD	1.812 (3.6)	260 (4.5)	531**(2.4)	61**(3.0)
≥1 villous adenoma	593 (1.2)	92 (1.6)	123**(0.6)	16**(0.8)
≥1 tubulovillous adenoma	10.396 (20.8)	1.455 (25.4)	3.404 (15.5)	408 (19.9)
≥1 10mm serrated polyp	10.912 (21.8)	1.564 (27.2)	4.018 (18.3)	458 (22.3)
≥1 traditional serrated adenoma	972 (1.9)	147 (2.6)	475 (2.2)	60 (2.9)
≥1 SSLD	1.831 (3.7)	736 (12.8)	799 (3.6)	239 (11.6)
≥1 CRC	2.765 (5.5)	436 (7.6)	726 (3.3)	100 (4.9)

*An advanced adenoma includes a tubulovillous adenoma, a villous adenoma, an adenoma with high-grade dysplasia (HGD), or an adenoma of at least 10mm. A high-risk adenoma includes adenomas with HGD, at least 5 low-risk adenomas, or an adenoma of at least 10mm. ** The difference between SSL and SSLD is not statistically significant

Table 2s Prevalence of sessile serrated lesions with dysplasia within FIT-positive screenees over time stratified by age and sex.

**SSLD Prevalence = % (95%CI)**	**<60**	**60-70**	**70+**
**2014 - 2018**	0.90 (0.80-1.01)	1.06 (1.00-1.12)	1.32 (1.24-1.41)
**Male**	0.95 (0.87-1.04)	1.08 (1.01-1.15)	1.27 (1.17-1.39)
**Female**	0.90 (0.75-1.09)	1.03 (10.95-1.11)	1.39 (1.26-1.53)
**2019 - 2023**	0.78 (0.72-0.84)	1.08 (1.02-1.14)	1.46 (1.38-1.54)
**Male**	0.83 (0.75-0.93)	1.04 (0.97-1.12)	1.34 (1.24-1.45)
**Female**	0.71 (0.62-0.80)	1.14 (1.05-1.23)	1.60 (1.47-1.73)

Table 3s Prevalence of sessile serrated lesions within FIT-positive screenees over time stratified by age and sex.

**SSL Prevalence = % (95%CI)**	**<60**	**60-70**	**70+**
**2014 - 2018**	8.20 (7.90-8.51)	7.79 (7.64-7.93)	7.50 (7.31-7.70)
**Male**	8.07 (7.68-8.48)	7.61 (7.43-7.80)	7.12 (6.88-7.38)
**Female**	8.38 (7.92-8.87)	8.04 (7.81-8.28)	8.03 (7.72-8.35)
**2019 - 2023**	10.4 (10.1-10.6)	11.5 (11.3-11.7)	11.6 (11.4-11.8)
**Male**	10.4 (10.1-10.7)	11.2 (10.9-11.4)	11.1 (10.8-11.4)
**Female**	10.3 (9.98-10.6)	12.0 (11.7-12.3)	12.3 (11.9-12.6)

Table 4s Overall rate of dysplasia over time.

	**2014**	**2015**	**2016**	**2017**	**2018**	**2019**	**2020**	**2021**	**2022**	**2023**
**SSLs (n)**	1.867	4.467	5.866	7.009	8.202	8.901	6.788	11.148	10.645	11.878
**SSLD (n)**	326	600	550	857	890	825	521	909	747	785
**Rate of dysplasia:**	**17.5%**	**13.4%**	**9.4%**	**12.2%**	**10.9%**	**9.3%**	**7.7%**	**8.2%**	**7.0%**	**6.6%**

Fig. 1s distribution of sessile serrated lesions throughout the colon versus sessile serrated lesions with dysplasia.

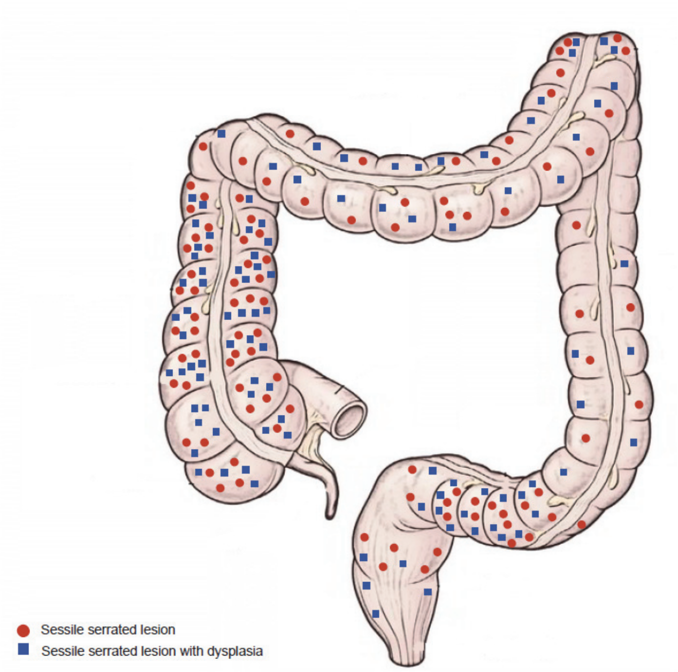

Supplement: Supplementary file 1 — Supplementary Material [file 10-1055-a-2847-3376_28538619.pdf]
